# Erjingwan Extracts Exert Antiaging Effects of Skin through Activating Nrf2 and Inhibiting NF-κB

**DOI:** 10.1155/2019/5976749

**Published:** 2019-05-02

**Authors:** Hairong Zhong, Choyoung Hong, Zhouxin Han, Seung Jin Hwang, Byunghyun Kim, Zihang Xu, Junmun Lee, Ki Hoon Kim, Mu Hyun Jin, Chunpu Zou

**Affiliations:** ^1^School of Basic Medical Science, Shanghai University of Traditional Chinese Medicine, Shanghai 201203, China; ^2^Oriental Herbal Medicine Research Team, LG Household & Health Care Co., Seoul 07795, Republic of Korea; ^3^Safety Researcher Lab, LG Household & Health Care Co., Seoul 07795, Republic of Korea

## Abstract

In oriental medicine, mixtures of medical plants are always used as prescriptions for diseases. Natural products extracted from herbs have great potential antiaging effects. Previous studies and clinical trials have shown several critical functions of Erjingwan (EJW), such as nourishing Yin, kidney tonifying and aging-resistance. We assumed that EJW extracts exerted the antiaging effects through nourishing Yin. We examined the antiaging effects of EJW extracts on healthy human skin by noninvasive measurements. Then we estimated the cell proliferation and DPPH radical scavenging rate. Western blotting analysis was used to determine the expressions of matrix metalloproteinase-1 (MMP-1), type I collagen (COL1A2), p-NF-*κ*B, NF-*κ*B, p-I*κ*B*α*, I*κ*B*α*, p-Nrf2, and HO-1. EJW extracts did not affect moisture content, TEWL and skin chroma, while it significantly improved skin glossiness and skin elasticity. Moreover, EJW extracts could downregulate the MMP1 expression and upregulate the COL1A2 expression. In addition, it promoted the Nrf2 pathway while it inhibited the NF-*κ*B pathway. With the application of cream containing EJW extracts, the skin aging state was significantly improved. Furthermore, in vitro studies showed that EJW extracts contributed to the repair of skin after injury. Taken together, the antiaging effects of EJW extracts were related to its antioxidant and anti-inflammatory abilities.

## 1. Introduction

Skin is the largest organ of the human body, and the functions of skin include protection, sensation, regulation of body temperature, secretion and excretion, absorption, and metabolism [1]. Skin aging often results in inordinate functions and destroyed structures, which are both affected by endogenous and exogenous factors. Aging-induced structural changes include flattened dermoepidermal connections, altered epidermal thickness, cell size and cell shape, dermal atrophy, reduced fibroblasts, mast cells and blood vessels, hair loss, and decreased hair pigmentation. Functional changes include cell turnover, wound repair, barrier function, secretion of sweat and sebum, and reduced synthesis of vitamin D. Endogenous factors include cellular metabolism, genetics, hormones, and metabolic processes, and exogenous factors include chronic exposures to light, pollution, ionizing radiation, chemicals, and toxins. No matter what kind of reasons, the representative external manifestations are wrinkles, spots, skin laxity, and roughness.

The mechanisms underlying the skin aging remain largely unexplored. It is well accepted that the main cause of skin aging and wrinkle formation is changes in collagen. About 90% of the proteins in dermis are collagen. Matrix metalloproteinases (MMPs) and MMP-1 subfamily may lead to collagen degradation in the skin [2]. Oxidative stress plays an important role in the progress of aging. Oxidative stress is the most harmful contributor to skin aging both internally and externally, and over production of reactive oxygen species (ROS) can induce the imbalance between free radical production and antioxidant defense. Previous studies have shown that the increased generation of ROS is significantly correlated with aging process [3]. Skin inflammation plays an important role in accelerated aging [4]. Two important transcription factors nuclear factor-*κ*B (NF-*κ*B) and nuclear factor (erythroid-derived 2)-like 2 (Nrf2) play fundamental roles in regulating cellular response to inflammation and oxidant stress. Nrf2 is considered as an auxiliary protective factor in the regulation of antioxidation, anti-inflammation and expressions of detoxification proteins [5]. IKK/NF-*κ*B inhibitors can decrease DNA damage that induces senescence and aging [6].

People always prefer healthy and younger-looking skin, which represents youth and beauty. There are many antiaging strategies, including preventive measures, cosmetic strategies, topical and systemic medications, and invasive procedures (such as chemical peeling, vitamin A, fluorouracil ointment, laser, intense pulsed light, skin filling operation, dermabrasion, cryotherapy, and photodynamic therapy) [7, 8]. Many antiaging skin care products have been developed in history, and over the years, advanced technologies and new theories have greatly contributed to the convenient and effective use of the skin care products. Many studies have shown that topical application of antiaging products can improve the appearance of skin, and topical application of vitamins, minerals, and botanical ingredients can improve fine wrinkles, freckles, and pigmentation. More and more attention has been paid to botanical ingredients due to their less toxicity and side effects [9].

Erjingwan (EJW) decoction is composed of* Lycium barbarum* and* Polygonatum sibiricum*, which is recorded in* General Medical Collection of Royal Benevolence*. EJW is a classic tonic formula, which can maintain youth and prevent decrepitude. It has been investigated that* Lycium barbarum* polysaccharide can decrease DNA damage through reducing oxidative stress [10]. Moreover, it can inhibit cell aging through the p53-mediated pathway [11].* Polygonatum sibiricum* is usually applied for Qi- or Yin-deficiency syndrome, and it has kidney-tonifying ability.* Polygonatum sibiricum* has been validated to have antioxidant effects, and it can enhance telomerase activity [12, 13]. Therefore, the compatibility of these two medicinal herbs contributes to the antiaging process.

Based on previous studies, we hypothesized that EJW extracts could be used in antiaging products. In the present study, we aimed to explore the mechanisms underlying the antiaging effects of EJW extracts and assess its antioxidant and anti-inflammatory abilities. We first confirmed the effectiveness of EJW extracts. Human foreskin fibroblasts (HFFs) were treated with H_2_O_2_ to induce the damage. Then some targets were determined to evaluate if EJW extracts could reverse such H_2_O_2_-induced damage. The DPPH free radical scavenging assay was conducted to validate the antioxidant ability of EJW extracts. Western blotting analysis was used to examine the expressions of MMP1 and COL1A2 as well as NF-*κ*B and Nrf2 pathways.

## 2. Materials and Methods

### 2.1. Clinical Trial

#### 2.1.1. Preparation of EJW Cream

The samples were produced by LG Household & Health Care Co., including cream containing EJW extracts with traditional Chinese medicine (TCM) nourishing Yin therapy (sample A) and cream without active ingredient (sample B).

Mix 100ml solvent (Water 88%, Butylene Glycol 10%, 1,2-Hexanediol 2%) with 10g Lycium Chinense Fruit Extract; 10g Polygonatum Sibiricum Extract; 10g Poria Cocos Extract; 10g Polygonum multiflorum, respectively. Then extract them at normal temperature for 3 days, and filter. Then mix the extractions together and filer again.

The components of the cream are listed in Table 5.

#### 2.1.2. Main Instruments

Following instruments were used in the present study, including human skin rapid optical imaging system PRIMOS Pico (GFMesstechnik GmbH, Germany), stratum corneum water component tester Corneometer (Courage & Khazaka, Germany), epidermal water loss rate tester Tewameter TM300 (Courage & Khazaka, Germany), CR-400 MINOLTA (KONICA, Japan), skin gloss tester GL200 (Courage & Khazaka, Germany), skin elasticity tester Dual Cutometer MPA580 (Courage & Khazaka, Germany), and balance, sense quantity 0.01 g (METTLER, Switzerland).

#### 2.1.3. Participants

The study was reviewed and approved by the National Quality Supervision and Inspection Center for Perfume and Perfume Cosmetics. Each participant provided written informed consent before any study-related examination or procedure was performed and the study adhered to the tenets of the Declaration of Helsinki. The participants were selected according to inclusion and exclusion criteria. The participants were required to voluntarily participate in the test and sign the informed consent: be healthy Chinese women, be between 30 to 45 years old, have obvious wrinkles or fine lines in the eye test area (grade 2-3 of crow's feet), keep the skin of arms dry (the base value of skin moisture in the forearm test area was between 15-45 a.u.), and refrain to use any cosmetics on the face from the night before the test. On the day of the test, participants should clean the face and forearms before the test; have no severe systemic diseases, no immunodeficiency or autoimmune diseases, and receive no skin cosmetology treatment and other tests; be not highly sensitive to body constitution, not sensitive to common cosmetics and not suffering from pollen allergy or other allergic reactions; ensure that no steroid drugs and immunosuppressive agents were used in the last month; ensure not to participate in other clinical studies in the last 3 months; agree to use photos of local skin and self-assessment results in the data; have no plan for outdoor activities or travel to the south and have no excessive sun exposure during the test period; ensure that the facial products of the same function other than the test products were not used during the test; ensure that the face was not treated by antiaging, whitening, grinding, or other cosmetic projects during the test period, and ensure that no drugs of antiaging and whitening effects were administered orally; well use the products, understand and fill in the project questionnaire and cooperate with the staffs; and have the chance to participate in further tests in the future. The exclusion criteria were as follows: people with allergic dermatitis or having a history of skin diseases (severe freckles, atopic skin diseases, psoriasis, severe acne dermatitis and so on) or people not suitable to participate in the project according to the clinical evaluation; people receiving treatment in dermatology, or people using skin, whitening and antiaging drugs within 1 month; the test area in the arms had a large area of birthmark, scratch, white spot, pigmented nevus and keloid; the face had a large area of birthmark, scratch, white spot, pigmented nevus and keloid; woman who was pregnant, breast-breeding and menopause, or who intended to conceive during the test; and woman who was not suitable as the object of this test.

A total of 22 healthy Chinese women (30-45 years old) were enrolled in this randomized, double-blind study, and 21 of them were recruited as participants, while one was excluded from subsequent analysis due to the agreement of withdrawal.

#### 2.1.4. Treatment and Test Requirements

The test environment required a temperature of 22± 2°C and a humidity of 40-60%.

Cream containing effective components (EJW extracts) was applied on the skin of the left face and one forearm for 4 weeks, while cream without such components was applied on the other side as a control.

During 4 weeks, participants were requested to clean the face and arms every morning and evening, and then 0.5 g of each cream was carefully spread to the face and forearm, followed by gentle massage until the cream was fully absorbed.

#### 2.1.5. Noninvasive measurements of the skin (Table 1)

#### 2.1.6. Test Schedule

**Figure 1:**
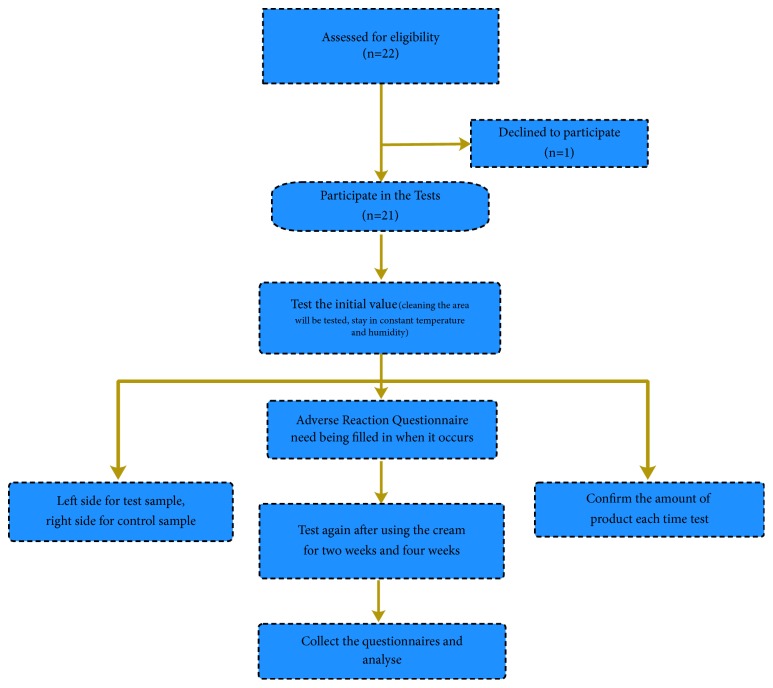
Study flowchart of the participants describing trial progress.

Tests were carried out according to a predesigned schedule as follows: test 0W (before use): 2016.11.29, Tuesday; test 2W: 2016.12.13, Tuesday; and test 4W: 2016.12.27, Tuesday). Specific implementation process is shown in Figure 1.

#### 2.1.7. Allergy Test (24 h Patch Test)

In order to examine whether the skin was allergic to the cream containing EJW extracts, 24 h patch test was performed. A total of 20 males without dermatosis were selected in the test. Briefly, 15 *μ*L test samples were spread on their backs for 24 h, and the degree of allergic reaction was observed after 2 h. Criteria of judgment were set as follows (Table 2).

### 2.2. In Vitro Study

#### 2.2.1. Cell Culture

HFF cells (Cellular Bank of Chinese Academy of Sciences) were maintained in Dulbecco's Modified Eagle's medium (DMEM, Gibco, USA) supplemented with 10% heat inactivated fetal bovine serum (Gibco, USA) and 1% penicillin streptomycin under standard conditions (37°C, 5% CO_2_).

#### 2.2.2. Cell Viability

Cell viability was assessed by Cell Counting Kit-8 (CCK-8, Byotime, China). Briefly, HFF cells were seeded into 96-well plates at a density of 4×10^3^/well and incubated under normal conditions for 24 h. The cells were divided into three groups, including control group, model group, and drug group. Moreover, the drug group was further divided into three subgroups with three different final concentrations (10, 100, and 200 *μ*g/mL). After 6 h, 10 *μ*L CCK-8 reagent was added to each well, and the mixture was incubated at 37°C for additional 2 h. Subsequently, the absorbance of each well was determined at a wavelength of 450 nm using the Synergy 2 Multi-Mode Microplate Reader (BioTek Instrument, Int., Winooski, VT, USA), and the cell viability was calculated with the formula as follows: cell viability (%) = average value of drug group/average value of control group.

#### 2.2.3. DPPH Free Radical Scavenging Assay

Stock solution of DPPH (D9132, Sigma Aldrich, USA) was prepared by adding 9.464 mg DPPH into 3 mL 80% absolute ethanol, yielding a concentration of 8 mM, and such stock solution was further diluted to 200 *μ*M when the test was conducted. Four groups were designed in this assay, and reagents in each group are listed in Table 3. Vitamin C (A610021, Sangon, China) was used as the positive control. Five concentrations (0.0625, 0.125, 0.25, 0.5, and 1 mg/mL) of EJW extracts were carried out in the test, and each concentration was conducted in triplicate.

After reagents were mixed well in 96-well plates, the mixture was incubated at 37°C for 30 min, and the absorbance was determined at a wavelength of 517 nm using Synergy 2 Multi-Mode Microplate Reader. Independent experiments were performed in triplicate. The inhibition rates were calculated with the formula as follows: Inhibition rate = (1-(B-C)/(A-D)) *∗* 100%. A represents the absorbance of total DPPH, B represents the absorbance of sample, C represents the absorbance of sample control, and D represents the absorbance of blank control. The graph was plotted by GraphPad prism 6.0, and then the IC50 value was calculated from the graph.

#### 2.2.4. Western Blotting Analysis

HFF cells in logarithmic growth were seeded into 6-well plates at a density of 1×10^5^ cells/well and incubated in the incubator for 24 h. Except for the control group, cells were incubated with 2 mL of 0.015 % H_2_O_2_ for 15 min. Culture medium in the model group was replaced by 2 mL fresh culture medium, while 2 mL of 250 *μ*g/mL EJW extracts was added to the drug group and incubated for 24 h and 48 h. Next, cells were lysed by 40 *μ*L RIPA buffer (Beyotime, China) on ice for 15 min, and the cell lysates were centrifuged at 12,000 rpm for 15 min at 4°C. Protein concentrations were determined by BCA method (Sangon, China). Equal amounts of proteins (15 *μ*g) were subjected to 10% sodium dodecyl sulfate-polyacrylamide gel electrophoresis (SDS-PAGE) and then transferred onto nitrocellulose membranes (GE). The membranes were blocked by Quick Block (Beyotime, China) for 10 min and then incubated with primary antibodies against COL1A2 (CY-5120, Abways, China), MMP1 (D220093, Sangon, China), p-NF-*κ*B (ser536) (11014, SAB, USA), NF-*κ*B (D220135, Sangon, China), p-I*κ*B*α* (ser32/ser36) (D155066, Sangon, China), I*κ*B*α* (D120138, Sangon, China), p-Nrf2 (S40) (CY-6573, Abways, China), or HO-1 (sc-136960, Santa Cruz, USA) at 4°C overnight. Subsequently, the blots were washed with TBST for three times and then incubated with corresponding HRP-conjugated secondary antibodies (1:5,000 dilution) at 37°C for 30 min. Immunoreactive bands were visualized with ECL reagents (Millipore, USA), and imaging densitometry was quantified using ImageJ (NIH, USA).

#### 2.2.5. qRT-PCR

HFF cells were seeded into 6-well plates at a density of 7×10^4^/well 24 h prior to different treatments. Moreover, 0.015% H_2_O_2_ was used to treat the model group and EJW group for 15 min. Then H_2_O_2_ solution was removed, and then HFF cells in the EJW group were further incubated with 200 *μ*g/mL EJW extracts for 48 h. Total RNA was extracted and then reversely transcribed into cDNA with PrimeScript RT MasterMix kit (Takara). qRT-PCR was performed using SYBR Premix Ex Taq kit (Takara) on LightCycler 96 SW 1.1. The relative expressions of target genes at the mRNA level were determined using the 2^-ΔΔCT^ method, and GAPDH was selected as the housekeeping gene. The primer sequences for target genes are listed in Table 4.

### 2.3. Statistical Analysis

All data were expressed as mean ± SEM and analyzed using SPSS19.0 (IBM). Comparisons between experimental groups were conducted using one-way ANOVA, while multiple comparisons were performed using the LSD method. Statistical significance was defined as P < 0.05 or P < 0.01.

## 3. Results

### 3.1. Antiaging Effects of EJW-Containing Cream

**Figure 2:**
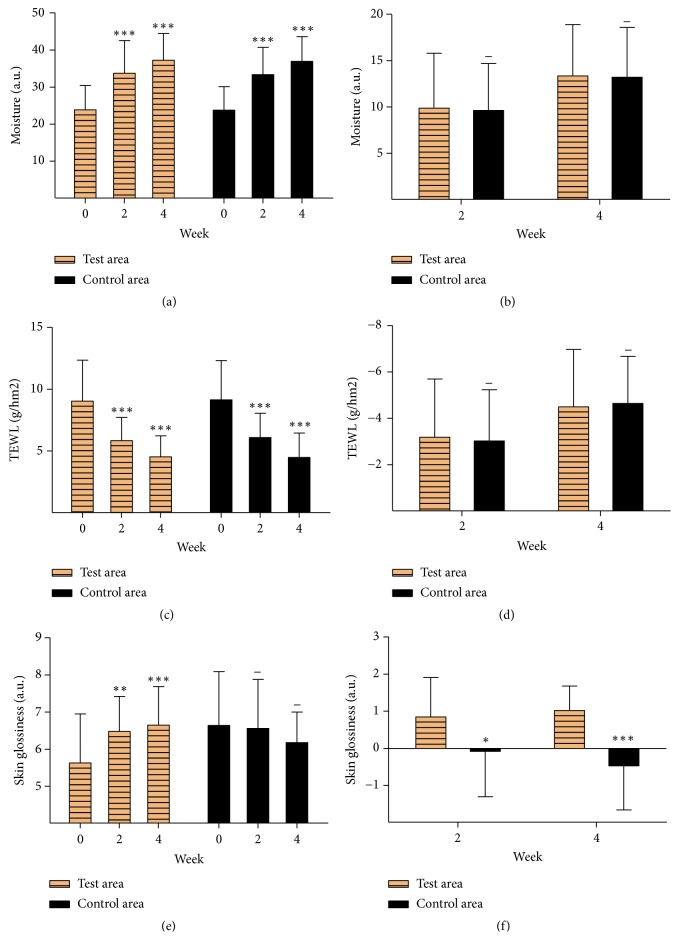
EJW extracts significantly improve skin aging. (a, b) Moisture content was measured by MPA10 Comeometer CM825. (c, d) MPA 10 Tewameter TM300 was used to measure the TEWL. (e, f) Skin glossiness was measured by MPA10 Skin-Glossmeter GL200. *∗* P<0.05; *∗∗* P<0.01; *∗∗∗* P<0.001; *∗∗∗∗* P<0.0001. Data were shown as mean ± SD.

Moisture content was determined using MPA10 Comeometer CM825. The higher value indicates the higher moisture content. Moisture content in the area applied with cream containing EJW extracts for 2 weeks and 4 weeks was significantly improved compared with their initial values as well as the control area (Figure 2(a)). No significant difference in the moisture increment was observed between the two groups, suggesting that the cream containing EJW extracts could not effectively increase the moisture of stratum corneum compared with the control sample (Figure 2(b)).

As an important indicator of skin function, transepidermal water loss rate (TEWL) is widely accepted as the most reliable method to examine the skin barrier function [14]. MPA 10 Tewameter TM300 was used to determine TEWL inside of forearms (left and right). Values of TEWL in both the control area and test area after 2 weeks and 4 weeks of treatment were significantly decreased compared with their initial values (Figure 2(c)). However, there was no significant difference in the reduction of TEWL between the two areas of forearms, indicating that the cream containing EJW extracts could not effectively decrease the TEWL of the forearm skin compared with the control sample (Figure 2(d)).

**Figure 3:**
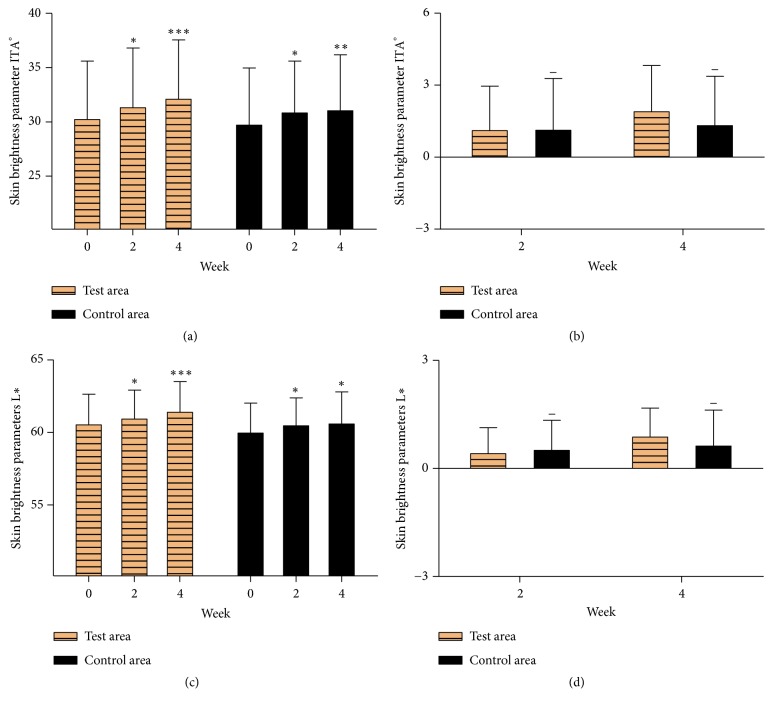
The skin chroma was evaluated by CR-400. *∗* P<0.05; *∗∗* P<0.01; *∗∗∗* P<0.001; *∗∗∗∗* P<0.0001. Data were shown as mean ± SD.

The measurements of skin chroma included L^*∗*^ (brightness of color) and ITA° (individual topology angle), which were evaluated by CR-400. The most widely used standard color system is Commission Internationale de l'Eclairage (CIE) Lab system, including L^*∗*^ (lightness value), a^*∗*^ (the amount of green or red), and b^*∗*^ (the amount of yellow or blue). The three axes record color in a 3-dimensional space [15]. The calculation formula of ITA° is tan^−1^⁡[(L^*∗*^ − 50)/b^*∗*^] × 180/*π*. ITA° is the angle between L^*∗*^ and b^*∗*^ in the 3-dimensional space, which has a positive correlation with L^*∗*^ and a negative correlation with b^*∗*^ [16]. The greater the values of L^*∗*^ and ITA° are, the brighter the skins are. Figures 3(a), 3(b), 3(c), and 3(d) show that skin brightness parameters L^*∗*^ and ITA° in both the test area and control area after 2 weeks and 4 weeks of treatment were significantly improved compared with their initial values. Meanwhile, no significant difference of skin chroma between the two areas was observed. This result suggested that the cream containing EJW extracts could not effectively improve the skin chroma compared with the control sample.

**Figure 4:**
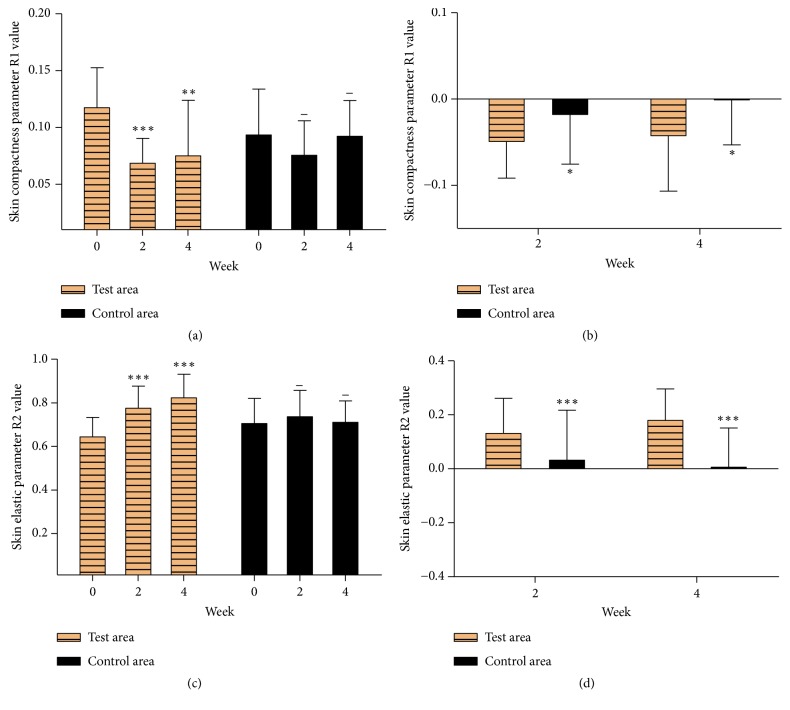
The skin elasticity was measured by Dual Cutometer MPA580. *∗* P<0.05; *∗∗* P<0.01; *∗∗∗* P<0.001; *∗∗∗∗* P<0.0001. Data were shown as mean ± SD.

Though there was no significant difference in moisture content, TEWL and skin chroma between the test area and control area, the skin glossiness in the area applied with cream containing EJW extracts for 2 weeks and 4 weeks was significantly improved compared with initial value, while it in the control area showed no difference (Figure 2(e)). Meanwhile, the skin glossiness in the test area was obviously higher than that in the control area (Figure 2(f)). This result indicated that EJW extracts played an important role in improving the skin glossiness compared with the control sample. Moreover, the skin elasticity test showed that the R1 value was significantly decreased and the R2 value was significantly increased only in the test area (Figures 4(a), 4(b), 4(c), and 4(d)). R1 value (skin firmness parameter) and R2 value (skin elasticity parameter) are two parameters of skin elasticity. The lower the R1 value is, the firmer the skin is. The higher the R2 value is, the resilient the skin is. In a word, the cream containing EJW extracts could strongly improve the skin elasticity compared with the control sample.

### 3.2. Allergy Test

**Figure 5:**
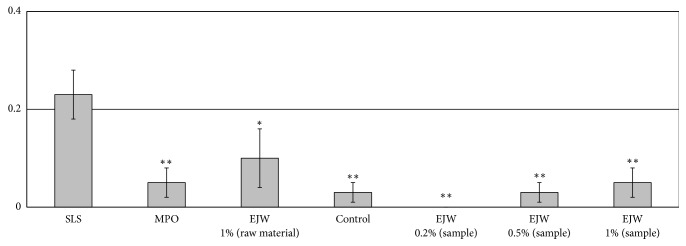
The formula of primary irritation index (PII) is [*∑*(score ×No. of responses)]/20 (No. of total subjects)×1 (total stimulus evaluation times). Criteria of judgment were set according to* CTFA/ICDRG guideline, text book of contact dermatitis *(Table 2). *∗* P < 0.05; *∗∗* P < 0.01. Data were shown as mean ± SD.

The sample groups included SLS (sodium lauryl sulfate), MPO (2-methyl- 1,3-propanediol), EJW (raw material), control, 0.2% EJW (sample), 0.5% EJW (sample), and 1% EJW (sample). SLS (Sodium lauryl sulfate or sodium dodecyl sulfate, SDS) is an anionic surfactant with the sample CH3(CH2)11SO4Na. It is a common component of many domestic cleansing, personal hygiene and cosmetic, pharmaceutical and food products. And SLS is used as a positive control in human skin primary irritation test. All the sample groups showed no reaction on the skin (Figure 5).

### 3.3. EJW Extracts Reverse the Damage of H2O2 to HFF Cells In Vitro

**Figure 6:**
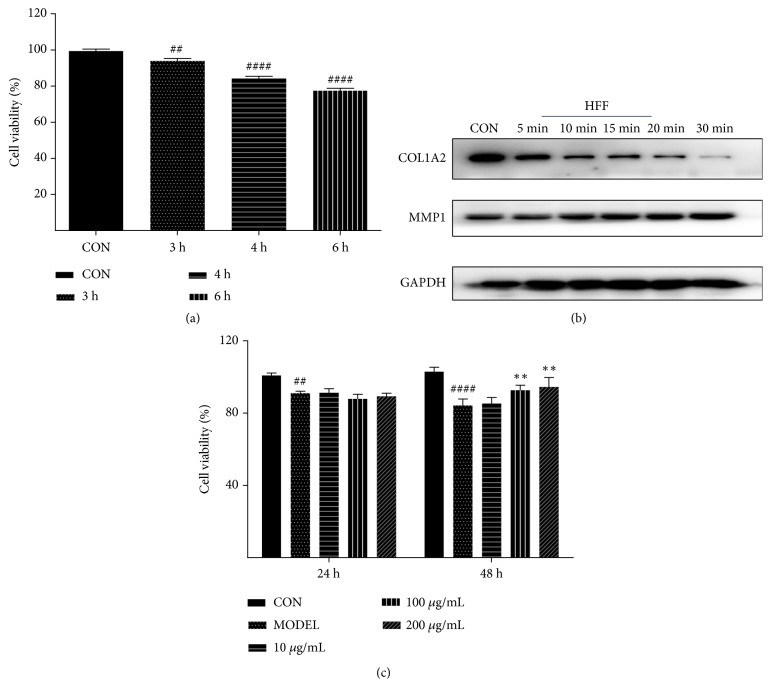
EJW extracts can improve the cell viability of H_2_O_2_-treated HFF cells. (a) Cell viability was significantly decreased by the treatment of 0.015% H_2_O_2_. (b) HFF cells were treated with 0.015% H_2_O_2_ for 5, 10, 15, 20, 30 min. (c) After exposure to 0.015% H_2_O_2_ for 15 min, HFF cells were incubated with EJW extracts (10, 100, 200 *μ*g/mL) for 24 h and 48 h. # indicates the significant difference between control group and model group, and *∗* indicates the significant difference between model group and EJW group. *∗* and # P<0.05;*∗∗* and ## P<0.01; *∗∗∗* and ### P<0.001; *∗∗∗∗* and #### P<0.0001. Data were shown as mean ± SD.

H_2_O_2_ was used as a stressor in the test. Briefly, 0.015% H_2_O_2_ was used to treat the HFF cells in vitro for different durations. The results showed that the cell viability was significantly decreased in a time-dependent manner (Figure 6(a)). Then we examined the expressions of MMP1 and COL1A2 at the protein level in HFF cells treated with 0.015% H_2_O_2_ for different durations, and our data showed that the expression of MMP1 was increased, while the expression of COL1A 2 was decreased (Figure 6(b)). Then HFF cells were incubated with 100 *μ*g/mL or 200 *μ*g/mL EJW extracts for 24 h and 48 h after H_2_O_2_ exposure, and we found that the cell viability of EJW-treated cells was significantly increased after 48 h (Figure 6(c)). Western blotting analysis also showed that the MMP1 expression was increased, the COL1A2 expression was decreased after H_2_O_2_ exposure, and 48 h exposure to EJW extracts could reverse such expression pattern (Figures 8(b) and 8(c)).

### 3.4. EJW Extracts Restore H_*2*_O_*2*_-Induced Damage through Modulating the Antioxidant and Anti-Inflammatory Pathways

**Figure 7:**
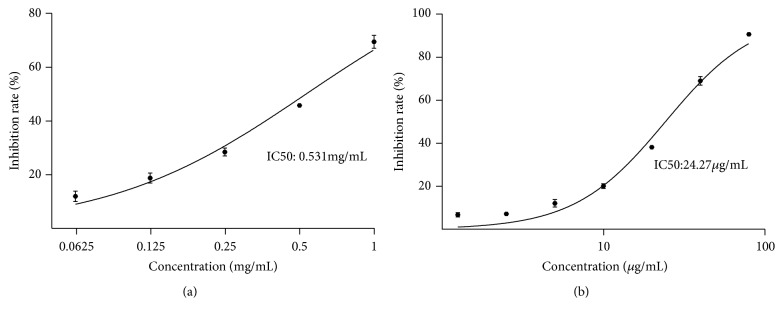
EJW extracts significantly inhibit the activity of DPPH. (a) DPPH inhibition rates of EJW extracts (0.0625, 0.125, 0.25, 0.5, and 1 mg/mL) were examined. Different concentrations of EJW extracts were incubated with DPPH for 30 min at 37°C. (b) Vitamin C (1.25, 2.5, 5, 10, 20, 40, and 80 *μ*g/mL) was used as the positive control (Table 3). Data were shown as mean ± SD.

**Figure 8:**
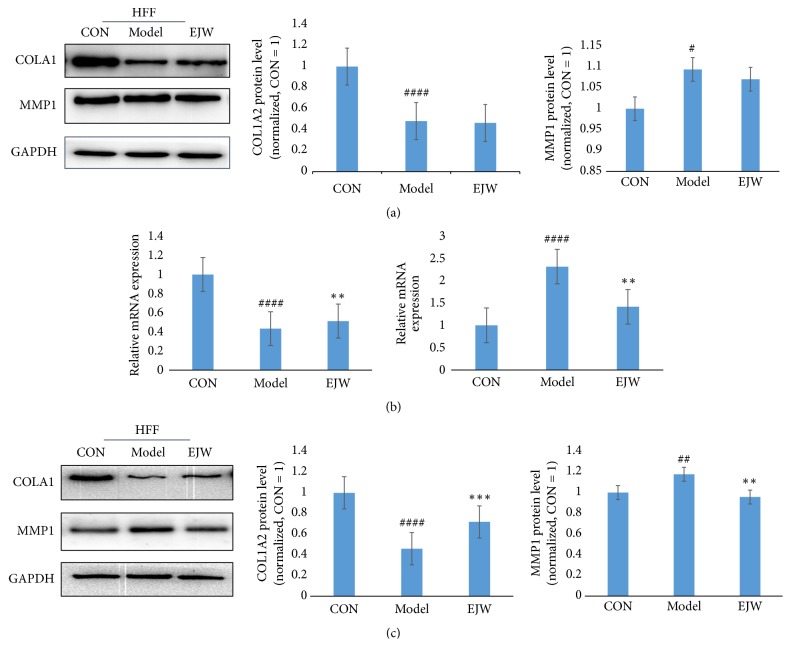
EJW extracts restore the expression of COL1A2 by reducing the MMP1 expression. (a) HFF cells were seeded into the 6-well plates and exposed to 0.015% H_2_O_2_ for 15 min. The expressions of COL1A2 and MMP1 at 24 h were examined by Western blotting analysis. (b, c) At 48 h, the expressions of COL1A2 and MMP1 were assessed by RT-qPCR and Western blotting analysis. The concentration of EJW was 200*μ*g/mL. # indicates the significant difference between control group and model group, and *∗* indicates the significant difference between model group and EJW group. *∗* and # P<0.05; *∗∗* and ## P<0.01; *∗∗∗* and ### P<0.001; *∗∗∗∗* and #### P<0.0001. Data were shown as mean ± SD.

**Figure 9:**
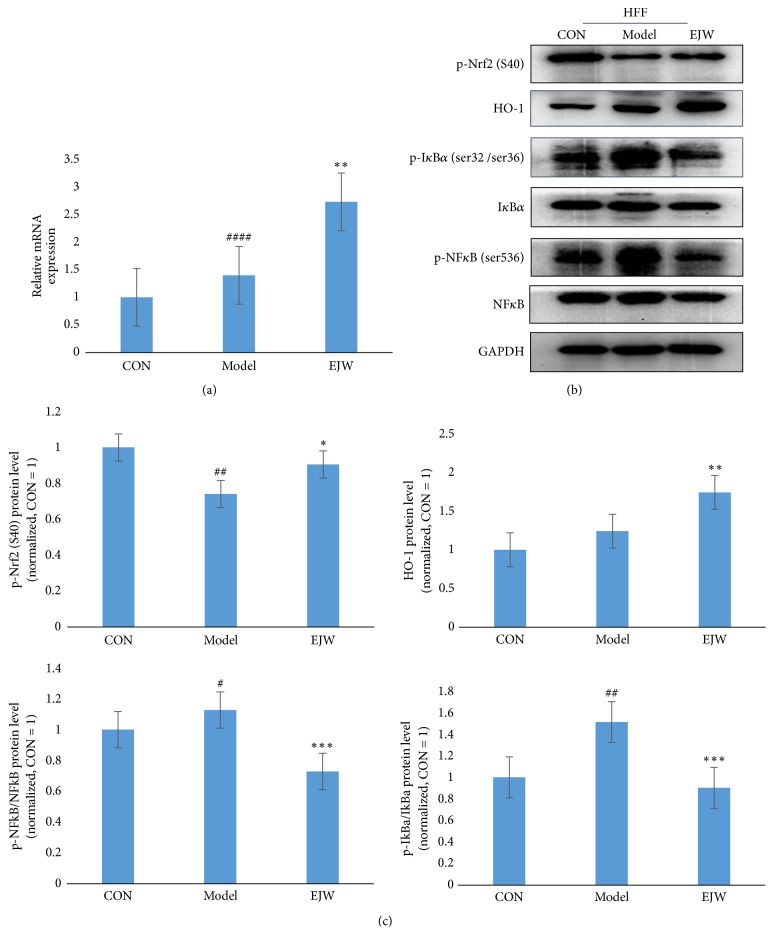
EJW extracts activate Nrf2 and inhibit NF-*κ*B to improve the aging HFF cells. (a, b) HFF cells were seeded into the 6-well plates and then exposed to 0.015% H_2_O_2_ for 15 min. The expression of HO-1 was detected by RT-qPCR, and the expressions of p-NF-*κ*B, NF-*κ*B, p-I*κ*B*α*, I*κ*B*α*, and p-Nrf2 were examined by Western blotting analysis. (c) Data were pooled from three independent experiments. # indicates the significant difference between control group and model group, and *∗* indicates the significant difference between model group and EJW group. *∗* and # P<0.05; *∗∗* and ## P<0.01; *∗∗∗* and ### P<0.001; *∗∗∗∗* and #### P<0.0001.

We showed that EJW extracts could significantly improve the skin glossiness and skin elasticity in healthy human skin and reverse the H_2_O_2_-induced damage of HFF cells in vitro. We next explored why EJW extracts had such effects. We first tested the DPPH inhibition ability of EJW extracts in vitro using Synergy 2 Multi-Mode Microplate Reader (BioTek Instrument, Int., Winooski, VT, USA).  Figure 7(a) shows that the EJW extracts significantly inhibited the DPPH activity, and the IC50 was 0.531 mg/mL. The results indicated that the antiaging effects of EJW extracts were related to the antioxidant ability. Therefore, we next assessed the Nrf2 pathway, an antioxidation pathway. As expected, the Nrf2 pathway was activated by the exposure to EJW extracts for 24 h. The expressions of p-Nrf2 and HO-1 were both significantly increased. Moreover, the H_2_O_2_-activated NF-*κ*B pathway was also downregulated by EJW extracts. Western blotting analysis showed that the expressions of p-NF-*κ*b and p-I*κ*B*α* were both significantly decreased (Figures 9(a), 9(b), and 9(c)).

## 4. Discussion

Herbal medicine has long been used in Asian countries, and herbal extracts can effectively reverse aging signs. Therefore, herbal ingredients have become a good choice for antiaging therapies [17]. Nowadays, a great deal of attention has been paid to herbal medicine worldwide. Topical application of herb extracts has attracted more and more attention due to its good skin care effect and low cost as well as noninvasive and less side effects. Hundreds of plant extracts from plant seeds, stems, skins, and fruits have been used in antiaging cosmetics [18]. Green tea extracts have shown antiphotoaging effect in clinical trial [19]. Algal extracts can resist UVB-induced skin lesion in BALB/C mice due to anti-inflammatory and antioxidative effects [20].

Herbal medicine has also long been used in China, and many herbal medicines are recorded to have antiaging effects and used as tonic formula in TCM therapies. EJW decoction is one of the classic decoctions. Firstly, our clinical trial validated the antiaging effects of EJW. Clinical trials included the evaluations of moisture content, TEWL, skin chroma, skin glossiness, and skin elasticity. They were all noninvasive detections of skin changes, which could be easily accepted by participants. The results indicated that EJW extracts could not increase the moisture content and keep water from running away (TEWL). Besides, it had no obvious whitening effects (L^*∗*^ and ITA°). On the contrary, skin glossiness and skin elasticity (R1 and R2) were both significantly improved. Therefore, dull and loose skin caused by aging could be improved by EJW extracts.

To explore the mechanism underlying antiaging effects of EJW extracts, H_2_O_2_ was used to induce the senescent state as an in vitro model of oxidative stress. Most contributing factors of skin aging, such as mitochondrial dysfunction, intracellular communication changes, genomic instability, cell senescence, and extracellular matrix (ECM) decomposition, are the consequences of oxidative processes. H_2_O_2_ is an ROS signaling agent, and generation of free radicals can lead to cell death, protein degradation, and inflammatory reaction [21]. To confirm the senescent state of HFF cells, we examined the cell viability by CCK-8 assay. The cell viability was significantly decreased upon the exposure to H_2_O_2_. Besides, the increased MMP1 expression and decreased COL1A2 expression also indicated the cell injury. Further studies revealed that EJW extracts could reverse the H_2_O_2_-induced injury. EJW extracts could promote the proliferation of HFF cells after H_2_O_2_ treatment, upregulate the expression of COL1A2 and downregulate the expression of MMP1, leading to reduced oxidative stress. Based on our findings, we deduced that the antiaging effects of EJW extracts were related to the antioxidative effects. Therefore, we conducted the DPPH free radical scavenging assay to validate such assumption.

Extensive studies have been focused on NF-*κ*B and Nrf2 pathways. Simultaneous regulation of NF-*κ*B and Nrf2 pathways plays an important role in many diseases. For example, Nrf2 improves lupus nephritis by inhibiting oxidative injury and negatively regulating the NF-*κ*B pathway [22]. The classical NF-*κ*B pathway is activated by stimuli, such as TNF-*α*, IL-1, H_2_O_2_, LPS, or microbial infection, further triggering the expressions of downstream genes, including various inflammatory cytokines and chemokines, after inducing the proteasome-mediated degradation of I*κ*B proteins [23]. NF-*κ*B pathway plays an important role in the occurrence and regulation of aging. It is not only activated in aging, but also directly involved in the development of aging-related diseases. For example, inhibition of NF-*κ*B can reduce cellular senescence and oxidative damage in mice or even extend the healthspan in elderly patients [24].

Nrf2 has detoxification and antioxidant effects, and the beneficial effects of Nrf2 have been previously reported in many diseases. UVB can induce more inflammation in the Nrf2 KO mice compared with the WT mice, while Nrf2 can protect the skin from UVB-induced inflammation and sunburn reaction [25]. Keap1 is an important negative regulator of Nrf2 in vivo. Under normal redox conditions, Nrf2 binds to typical Nrf2 repressor protein Keap1 in cytoplasm and is easily degraded by ubiquitin-proteasome pathway. However, Nrf2 dissociates from Keap1 when exposed to stressors or inducers, moves to the nucleus, binds to the ARE, and triggers the expressions of Nrf2-regulated genes. Therefore, the expressions of multiple antioxidant enzymes, such as superoxide dismutase (SOD), and biphase detoxifying enzymes, such as heme oxygenase 1 (HO-1), glutathione S transferase (GSTs) and NAD(P)H quinone oxidoreductase 1 (NQO1), begin to be upregulated [26–28]. HO-1 acts as a rate-limiting enzyme for heme degradation in all Nrf2-regulated enzymes, and it has antioxidant and anti-inflammatory, immunomodulatory and antiapoptotic effects. Once Nrf2 binds to ARE on the HO-1 promoter, HO-1 can be induced in many cells to exert its own defensive effect [5].

Moreover, there is an interaction between Nrf2, NF-*κ*B, and inflammation [29]. NF-*κ*B can downregulate the Nrf2 pathway, while the Nrf2-regulated gene HO-1 can inhibit NF-*κ*B activation. NF-*κ*B blocks the binding of CREB-binding protein (CBP) to Nrf2 or promotes the interaction of HDAC3 with CBP or MafK [30, 31]. Our results confirmed that EJW extracts might promote the Nrf2 pathway and inhibit the NF-*κ*B pathway, contributing to restoration of the redox homeostasis. EJW extracts possessed anti-inflammatory activities and excellent ROS-scavenging abilities. This could explain why EJW exhibited great antiaging effects on skin. However, there was a complex link between ROS, aging and lifespan [26]. On one hand, low levels of oxidative stress are found to be good for longevity and health [32]. On the other hand, it is difficult to quantify the experimental ROS level. Generally, the results of the experiments may be easy to deviate and produce unexpected results [21]. Therefore, completely scavenging free radicals may not be a good choice. In the present study, the successful establishment of H_2_O_2_-treated model was confirmed by examining cell viability and the expressions of MMP1 and COL1A2. EJW extracts might lower ROS to standard levels through the regulation of oxidation-reduction balance, and the specific mechanism needs to be further explored.

According to the aging theory of TCM, aging is a result of decline in vital energy. Therefore, maintaining the vital energy is the most important aspect in antiaging strategies. Yin essence is the most important basic substance of body, which is important for the body to maintain the balance of Yin and Yang. As two sides of a coin, a balance between Yin and Yang is similar to the balance between antioxidant and oxidant, while Yin represents antioxidant and Yang represents oxidant. Over oxidative stress is the imbalance of Yin and Yang. In TCM theory, inflammation is caused by the excessive Yang, which can be released by Yin-tonifying or cleared up by cold-cool drug [33]. The antiaging herbs have some common properties. For example, they can supplement the vital energy to the body, intervene with disease, and have multitargets to regulate the biological process [34]. One important future direction of the antiaging effects of EJW extracts is to find the target groups for Yin or Yang.

## 5. Conclusions

Taken together, our results indicated that EJW extracts could significantly improve the skin aging state, including skin glossiness and skin elasticity. EJW extracts might be involved in redox regulation by activating Nrf2 pathway and inhibiting NF-*κ*B pathway, leading to scavenging of free radicals as well as MMP1 downregulation and COL1A2 upregulation. The antiaging effect was related to the balance between Yin and Yang, where redox homeostasis was one of the aspects. In conclusion, like many other herbal medicines, EJW extracts could be applied in the antiaging products.

## Figures and Tables

**Table 1:** Observe projects and test site.

Observe and test projects	Test area and control area
1. Moisture content (MPA10 Comeometer CM825)	Inside of forearms
2. Trans epidermal water loss rate (MPA10 Tewameter TM300)	Inside of forearms
3. Skin chroma (CR-400)	Joint of the eye midline and nose wing
4. Skin glossiness (MPA10 Skin-Glossmeter GL200)	Cheekbones
5. Skin elasticity (Dual Cutometer MPA580)	Corner of the eyes

**Table 2:** CTFA/ICDRG guideline, textbook of contact dermatitis.

Reaction	Score	Symptoms and criteria of judgment
-	0	Adiaphoria
+/-	0.5	Light erythema
+	1.0	Obvious erythema
++	2.0	Severe erythema with swelling
+++	3.0	Severe erythema with vesicles and swelling

**Table 3:** Reagents in different groups.

Groups	Sample (*μ*L)	200 *μ*M DPPH (*μ*L)	80% absolute ethanol (*μ*L)
A	-	200	50
B	50	200	-
C	50	-	200
D	-	-	250

**Table 4:** The primer sequences for target genes.

MMP1 forward	5′-GAGCAGATGTG-GACCATGCC-3′
MMP1 reverse	5′-TGGGCCTGGTTGAAAAGCAT-3′
COL1A2 forward	5′-G-TGCTACTGGTGCTGCCG-3′
COL1A2 reverse	5′-CACACCCTGGGGACCTTCAG-3′
HO-1 forward	5′-GGCCATGAACTTTGTCCGGT-3′
HO-1 reverse	5′-TCAGTGCCCACGGTAAGGAA-3′
GAPDH forward	5′-GGCACAGTCAAGGCTGAGAATG-3′
GAPDH reverse	5′-ATGGTG-GTGAAGACGCCAGTA-3′

**Table 5:** The components of the cream.

Components
WATER
CYCLOHEXASILOXANE
HYDROGENATED POLYDECENE
DIPROPYLENE GLYCOL
GLYCERIN
MACADAMIA TERNIFOLIA SEED OIL
TRIETHYLHEXANOIN
BIS-PEG-18 METHYL ETHER DIMETHYL SILANE
1,2-HEXANEDIOL
STEARYL ALCOHOL
GLYCERYL STEARATE
PEG-40 STEARATE
PENTAERYTHRITYL TETRAETHYLHEXANOATE
CETEARYL ALCOHOL
STEARIC ACID
PEG-100 STEARATE
SORBITAN STEARATE
HYDROGENATED LECITHIN
CETEARYL OLIVATE
SORBITAN OLIVATE
DIMETHICONE
DIMETHICONE/VINYL DIMETHICONE CROSSPOLYMER
LACTOBACILLUS/SOYBEAN FERMENT EXTRACT
SACCHAROMYCES/POTATO EXTRACT FERMENT FILTRATE
SACCHAROMYCES/BARLEY SEED FERMENT FILTRATE
PANTHENOL
XANTHAN GUM
ACRYLATES/C10-30 ALKYL ACRYLATE CROSSPOLYMER
CARBOMER
TROMETHAMINE
TRISODIUM EDTA
FRAGRANCE
LYCIUM CHINENSE FRUIT EXTRACT
POLYGONATUM SIBIRICUM EXTRACT
POLYGONUM MULTIFLORUM ROOT EXTRACT
PORIA COCOS EXTRACT
SOPHOCARPINE

## Data Availability

The data used to support the findings of this study are available from the corresponding author upon requset.
